# Genome-Wide Analysis and Heavy Metal-Induced Expression Profiling of the HMA Gene Family in *Populus trichocarpa*

**DOI:** 10.3389/fpls.2015.01149

**Published:** 2015-12-23

**Authors:** Dandan Li, Xuemei Xu, Xiaoqing Hu, Quangang Liu, Zhanchao Wang, Haizhen Zhang, Han Wang, Ming Wei, Hanzeng Wang, Haimei Liu, Chenghao Li

**Affiliations:** ^1^State Key Laboratory of Tree Genetics and Breeding, Northeast Forestry UniversityHarbin, China; ^2^Library of Northeast Forestry UniversityHarbin, China

**Keywords:** *Populus*, P_1B_-type ATPases, HMA gene, heavy metal stress, phytoremediation

## Abstract

The heavy metal ATPase (HMA) family plays an important role in transition metal transport in plants. However, this gene family has not been extensively studied in *Populus trichocarpa*. We identified 17 HMA genes in *P. trichocarpa* (*PtHMA*s), of which *PtHMA1–PtHMA4* belonged to the zinc (Zn)/cobalt (Co)/cadmium (Cd)/lead (Pb) subgroup, and *PtHMA5–PtHMA8* were members of the copper (Cu)/silver (Ag) subgroup. Most of the genes were localized to chromosomes I and III. Gene structure, gene chromosomal location, and synteny analyses of *PtHMA*s indicated that tandem and segmental duplications likely contributed to the expansion and evolution of the *PtHMA*s. Most of the HMA genes contained abiotic stress-related *cis*-elements. Tissue-specific expression of *PtHMA* genes showed that *PtHMA1* and *PtHMA4* had relatively high expression levels in the leaves, whereas Cu/Ag subgroup (*PtHMA5.1- PtHMA8*) genes were upregulated in the roots. High concentrations of Cu, Ag, Zn, Cd, Co, Pb, and Mn differentially regulated the expression of *PtHMAs* in various tissues. The preliminary results of the present study generated basic information on the HMA family of *Populus* that may serve as foundation for future functional studies.

## Introduction

One of the negative effects of industrialization is heavy metal pollution, which is deleterious not only to the environment but also to human health. Heavy (transition) metals cause toxicity at the cellular level by binding to sulfhydryl groups in proteins and inhibiting enzyme activity or protein function. Exposure to heavy metals may also induce deficiencies in other essential ions by disrupting cellular transport processes and causing oxidative damage (Williams and Mills, 2005). Plants have evolved regulatory mechanisms on heavy metal ion toxicity tolerance and ensure an adequate supply of essential nutrients. Membrane transport proteins play an important role as scavengers of heavy metals. In recent years, progress in the development of genetic and molecular techniques has helped with in identification of various gene families that function in metal transportation (Williams and Mills, 2005).

The P_1B_-type ATPase, also known as the heavy metal ATPases (HMAs), which belongs to the large P-type ATPase family, plays an important role in transition metal transport in plants (Mills et al., 2003; Gravot et al., 2004; Hussain et al., 2004). It can selectively absorb and transport essential metal ions (Cu^2+^, Zn^2+^, and Co^2+^) for plant growth and development, as well as participate in the distribution of non-essential heavy metal ions (Cd^2+^ and Pb^2+^). The typical P_1B_-ATPase consists of approximately 6–8 transmembrane helices, a soluble nucleotide binding domain, phosphorylation domain, and a soluble actuator domain. The interactions of these three domains play an important part in the mechanism (Smith et al., 2014). In addition, both sides of the N-terminal and C-terminal metal binding sites comprise metal binding domains that interact with and bind specific metal ions (such as Cd^2+^/Pb^2+^). P_1B_-type ATPase is also located in this site, which is a heavy metal-associated regulatory domain as well (Williams and Mills, 2005; Arguello et al., 2007).

HMAs provides metal resistance, absorption, and transport. Based on metal substrate specificity, HMAs can be clustered into two major phylogenetic subclasses, namely, the Cu/Ag P_1B_-ATPase group and the Zn/Co/Cd/Pb P_1B_-ATPase group (Axelsen and Palmgren, 1998). The HMA proteins have been studied at the genomic scale in *Arabidopsis thaliana* and rice (*Oryza sativa*). There are eight and nine members of P_1B_-ATPase in *A. thaliana* and *O. sativa*, respectively (Williams and Mills, 2005). *AtHMA1–4* in *A. thaliana* and *OsHMA1–3* in *O. sativa* belong to the former group, whereas *AtHMA5–8* and *OsHMA4–9* belong to the latter group (Williams and Mills, 2005). The *Arabidopsis* disruption mutant of *AtHMA1* is sensitive to high concentrations of Zn (Moreno et al., 2008). Overexpression of *AtHMA3* induces Cd accumulation in plants, with a 2.5- and 2-fold higher in the roots and shoots, respectively (Morel et al., 2009). *AtHMA4* knockout plants are more sensitive to excess Zn(II) and Cd(II), and accumulate Zn less in shoots and more in roots than wild-type plants (Verret et al., 2004; Mills et al., 2005). High Cu concentrations elevate the expression of *OsHMA5* (Deng et al., 2013). Some members of P_1B_-type ATPases were identified in other plant species, including barley, wheat, *Thlaspi caerulescen*s, and *A. halleri* (Deng et al., 2013).

Early phytoremediation studies have shown that *Populus trichocarpa*, a fast-growing tree species, could be a suitable candidate for the treatment of heavy metal-polluted soils (Cunningham, 1996). Poplars transpire large amounts of water, thereby reducing contaminants from soil and water (Zacchini et al., 2009). Because of this feature, *Populus* spp. has attracted the attention of researchers working on remediation of heavy metal-contaminated soils. The completion of the *P. trichocarpa* genome sequence in 2006 presents an excellent opportunity to investigate the metal transporter families in this plant species (Tuskan et al., 2006). Although HMA genes have been extensively studied in various plants, including *Arabidopsis*, rice, and barley, investigations on the HMA gene family in *Populus* are limited. To compare the mechanisms on metal phytoremediation between woody and herbaceous plants that maintain different life cycles and considering the importance of the HMA gene family in plant responses to heavy metal stresses, we investigated *HMA* genes in *Populus*. In the present study, we performed a genome-wide analysis of the *P. trichocarpa* HMA gene family, its phylogenetic analysis, chromosomal distribution, and expressional analysis. We identified a total of 17 HMA genes in *P. trichocarpa*. Quantitative real-time RT-PCR (qRT-PCR) showed that the HMA genes in *Populus* were differentially regulated by excessive Cu, Ag, Zn, Cd, Co, Pb, and Mn stress. The results provide insights for future investigations into the roles of these candidate *HMA* genes in responds to metal stress in *Populus*.

## Materials and methods

### Identification of HMA genes in populus

We used Pfam (http://pfam.sanger.ac.uk/) to query HMA genes in the *P. trichocarpa* genome. All *Populus* HMA genome sequences could be directly downloaded from Phytozome (http://www.phytozome.net/) and NCBI (http://www.ncbi.nlm.nih.gov/). The downloaded HMA candidates sequences were analyzed manually using the SMART (http://smart.embl-heidelberg.de/) database to verify the presence of the HMA domain (Letunic et al., 2004). *A. thaliana* and *O. sativa* HMA subfamily sequences were downloaded from the *Arabidopsis* Information Resource (TAIR, http://www.arabidopsis.org/index.jsp, release 10.0) and Rice Information Resource (http://rice.plantbiology.msu.edu/index.shtml), respectively.

### Phylogenetic analysis

Multiple sequence alignment of the full-length protein sequences was performed using the ClustalX (version 1.83) program (Thompson et al., 1997) and manually adjusted using the BioEdit 7.1 software (Hall, 1999). A phylogenetic tree was constructed using the neighbor-joining (NJ) method, with a bootstrap test performed using 1000 iterations in MEGA5 (Tamura et al., 2007). Bootstrap analysis with 1000 replicates was used to evaluate the significance of the nodes. Gene clusters referred to the homologs within the three species (*P. trichocarpa, A. thaliana*, and *O. sativa*) that were identified based on the NCBI database (http://www.ncbi.nlm.nih.gov/).

### Chromosomal location and subcellular location of HMA genes

The chromosomal localization of all genes was determined and plotted with PopGenIE (http://www.popgenie.org/). Genes separated by =5 gene loci within a range of 100-kb distance were considered tandem duplicates (Hu et al., 2010). Identification of homologous chromosome segments resulting from whole-genome duplication events was conducted as described by Tuskan et al. (2006) WoLF PSORT (http://wolfpsort.org/) was used to predict the subcellular localization of the *HMA* proteins (Horton et al., 2007).

### Exon-intron structure and motif analysis

The online software Gene Structure Display Server (GSDS; Guo et al., 2007) was used to generate the exon/intron organization of HMA genes by comparing the cDNAs to its corresponding genomic DNA sequences in Phytozome (http://www.phytozome.net/search.php). The Multiple Expectation Maximization for Motif Elucidation (MEME) system (Version 4.9) was used to investigate conserved motifs for each HMA gene (Bailey et al., 2006).

### Promoter *cis*-element identification and microarray analyses

Promoter sequences, located 2000 kb upstream of the translation start site, were searched using NCBI, and were analyzed in the Plant Care database (http://bioinformatics.psb.ugent.be/webtools/plantcare/html/) (Lescot et al., 2002). To obtain the expression of each gene in different tissues and organ, we searched the microarray dates from PopGenIE v2.0 (http://popgenie.org/gp) in the NCBI Gene Expression Omnibus (GEO) database using the series accession number GSE6422.

### Plant materials and treatment

Clonally propagated *P. trichocarpa* genotype Nisqually-1 was cultured in half-strength Murashige and Skoog medium under long-day conditions (16 h light/8 h dark) at 23–25°C. Plants were exposed to the following: 100 μM CdCl_2_, 100 μM CuSO_4_,60 μM MnSO_4_, 750 μM Pb(NO_3_)_2_, 200 μM ZnSO_4_, and 1 mM AgNO_3_, and 50 μM CoCl_2_. Each treatment lasted for 0, 1, 3, and 10 h, and samples were collected at each time point. Three biological replicates were prepared for each stress treatment. All samples were immediately frozen in liquid nitrogen and stored at −80°C until analysis. Non-treated plants were used as control.

### RNA isolation and real-time quantitative PCR

Total RNA was extracted from the roots, stems, and leaves using the CTAB method (Jaakola et al., 2001). Prior to cDNA synthesis, RNA was treated with RQ1 RNase-free DNase (Promega, Madison, WI, USA), according to the manufacturer's instructions to rule out DNA contamination. First-strand cDNA synthesis was performed with the PrimeScript™ RT reagent kit (Perfect Real Time; Takara, Dalian, China). Primer Premier 5 was used to design gene-specific primers for all HMA genes based on its corresponding CDS. BLAST was used to check each primer to determine its specificity for the respective gene, and was further confirmed by melting curve analysis of real-time PCR reactions. All primer sequences are presented in Table S1.

SYBR Premix Ex Taq II (TaKaRa, Dalian, China) was used to perform qRT-PCR to determine the transcript levels of HMA genes in *P. trichocarpa* under different metal stresses. Reactions were prepared in a total volume of 20 μL that contained the following: 2 μL of template, 10 μL of 2 × SYBR Premix, 6 μL of ddH_2_O, and 1 μL of each specific primer, prepared to a final concentration of 10 μM. Three technical replicates were performed for each sample to ensure the accuracy of the results. The *P. trichocarpa actin* gene (GenBank Acc. No.: XM_002298674) was used as reference gene (An et al., 2011). A relative quantification method (2^−ΔΔCT^) was used to evaluate quantitative variation among replicates. The PCR conditions and relative gene expression calculations were as previously described (Zhang et al., 2013).

## Results

### Identification of HMA genes in *Populus*

We identified a total of 17 HMA genes (Table 1), of which *PtHMA4(1), PtHMA4(2), PtHMA4(3), PtHMA4(4), PtHMA4(5)* could be attributed to alternative splicing of mRNA with *PtHMA4*, which generates a variety of mature RNA transcripts that produce protein isoforms. The identified *PtHMA* genes encoded proteins that varied in length from 626 to 1228 amino acids (AA), and with an average of 928 AA. We used WoLF PSORT to predict the location of the proteins and noticed that most *PtHMA* genes were predicted as plasma membrane proteins, except *PtHMA1* and *PtHMA5.1*, which were located in the cytoplasm (Table 1).

**Table 1:** **The HMA gene family in ***Populus trichocarpa*****.

**Gene name**	**Accession number**	**NCBI Locus ID**	**Length(AA)**	**Protein subcellular Localization prediction**
*PtHMA1*	POPTR_0007s10480	XM_002310080.2	833	cyto^a^
*PtHMA4*	POPTR_0006s07650	XM_002327518.1	1228	plas
*PtHMA4(1)*			873	
*PtHMA4(2)*			1126	
*PtHMA4(3)*			1169	
*PtHMA4(4)*			1170	
*PtHMA4(5)*			1193	
*PtHMA5.1*	POPTR_0003s12580	XM_002303545.1	983	cyto
*PtHMA5.2*	POPTR_0001s09210	XM_002299504.2	985	plas
*PtHMA5.3*	POPTR_0003s12570	XM_002303544.2	987	plas
*PtHMA5.4*	POPTR_0001s05650	XM_002299198.1	974	plas
*PtHMA5.5*	POPTR_0003s20380	XM_006385962.1	626	plas
*PtHMA6.1*	POPTR_0001s21290	XM_002299649.2	807	plas
*PtHMA6.2*	POPTR_0003s01850	XM_002304058.2	808	plas
*PtHMA7.1*	POPTR_0001s15900	XM_002326412.1	1010	plas
*PtHMA7.2*	POPTR_0003s07330	XM_002303313.1	1008	plas
*PtHMA8*	POPTR_0018s08380	XM_006371981.1	889	plas

a *plas, plasma membrane; cyto, cytoplasm*.

### Phylogenetic, gene structural, and conserved motif analyses of *PtHMA* genes

**Figure 1:**
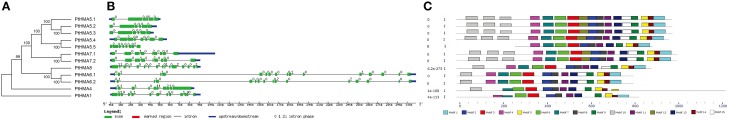
**Phylogenetic relationships, gene structure, and motif composition of HMA genes in ***P. trichocarpa***. (A)** Multiple alignments of 12 full-length amino acids of HMA genes were executed by ClustalX 1.83, and the phylogenetic tree was constructed using MEGA 5.0 using the neighbor-joining (NJ) method with 1000 bootstrap replicates. **(B)** Exon/intron structures. Exons and introns are represented by green boxes and black lines, respectively. **(C)** Schematic representation of the conserved motifs elucidated by MEME. Each motif is represented by a number in the colored box. The black lines represent the Non-conserved sequences.

To examine the phylogenetic relationships among the *Populus* HMA domain proteins in *Populus*, an unrooted phylogenetic tree was constructed from alignments of the full-length HMA sequences (Figure 1A). We classified 12 HMA genes into two subgroups, namely, Zn/Co/Cd/Pb (*PtHMA1–4*) and Cu/Ag (*PtHMA5–8*). Near 8000 pairs of paralogous genes are distributed in the *Populus* genome (Guo et al., 2009). By phylogenetic analysis, four paralogous pairs among the 12 *Populus* HMA genes were identified (Figure S1).

A comparison of the exon/intron organization of the coding sequences of individual *PtHMA* genes showed that most closely related members shared similar exon/intron structures either according to the number of introns or exon length. These results were consistent with the characteristics defined in the above phylogenetic analysis (Figure 1A). The coding sequences of most of the HMA genes were disrupted by introns, with the number of introns ranging from 5 to 16. *PtHMA8* exhibited the largest number of introns (16), followed by *PtHMA6.1* and *PtHMA6.2* (15) and *PtHMA1* (12). Other genes had less than 10 introns (Figure 1B).

Table S2 presents the conserved motifs that were shared among related proteins within the family and 15 distinct motifs (Table S2). The number of HMA motifs in *Populus* HMA proteins and the spacing of amino acids between adjacent HMA motifs varied, which was similar to that observed in *Arabidopsis* and rice HMA proteins. Most of the closely related members in the phylogenetic tree shared common motifs, and all HMA genes contained common motifs, except for motifs 1, 4, 10, 12, and 15 (Figure 1C). All HMA family members contained motif 10, except *PtHMA1*, thereby indicating that at least one motif was lost in this gene during its divergence from a common ancestor. Moreover, differences in gene organization and motif composition among related members suggested that these genes may be functionally divergent.

### Comparative analysis of the HMA genes in *Populus, Arabidopsis*, and rice

**Figure 2:**
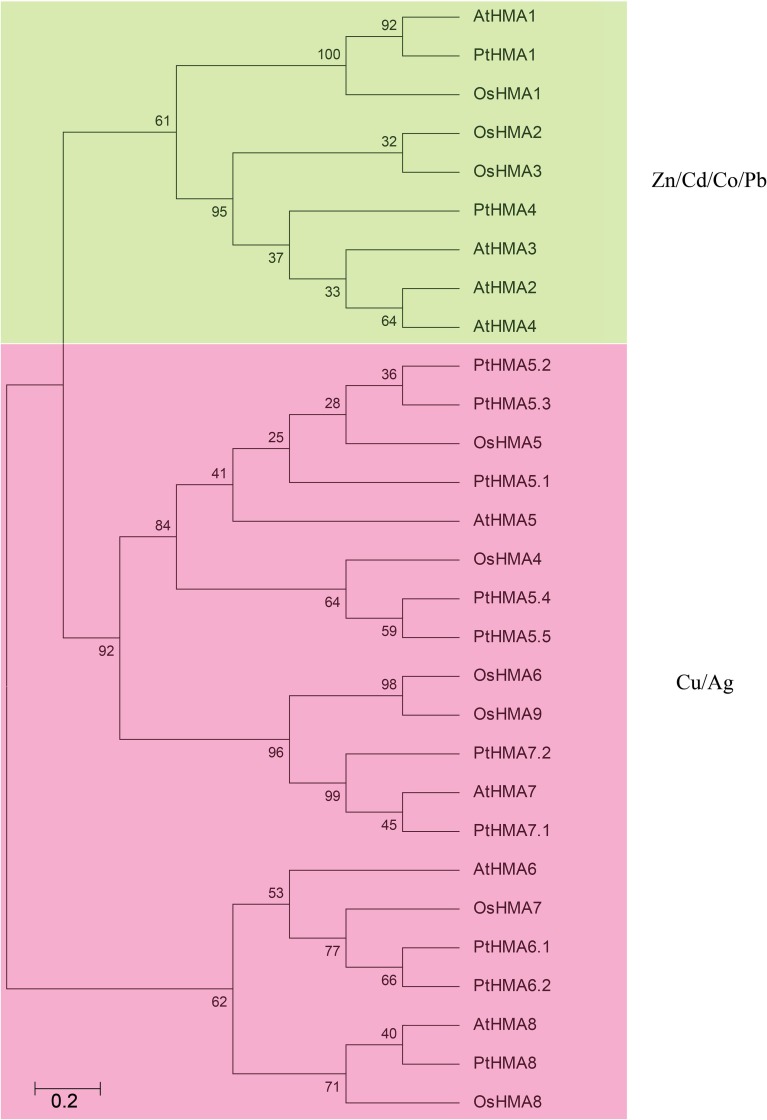
**All HMA proteins of ***P. trichocarpa*** (12), A. ***thaliana*** (8), and ***O. sativa*** (9) were divided into two distinct subfamilies**. The phylogenetic tree was constructed using MEGA 5.0 using the neighbor-joining (NJ) method with 1000 bootstrap replicates. Green represents the Zn/Cd/Co/Pb subgroup; pink indicates the Cu/Ag subgroup.

We examined the phylogenetic relationship among the HMA domain proteins in *Populus* (12 genes), rice (9 genes), and *Arabidopsis* (8 genes) that were constructed from alignments of full-length HMA protein sequences (Figure 2). The tree topologies produced by the three algorithms were largely comparable, and there were only minor modifications at interior branches (data not shown). Therefore, we further analyzed only the NJ phylogenetic tree in the present study. The HMA gene family contains different numbers and types of HMAs. There are eight and nine members of P_1B_-ATPase in *A. thaliana* and rice, respectively (Williams and Mills, 2005). These were then further divided into two groups: Zn/Cd/Co/Pb and Cu/silver (Ag) transporters (Axelsen and Palmgren, 1998). *AtHMA1–AtHMA4* and *OsHMA1–OsHMA3* belonged to the Zn/Co/Cd/Pb subgroup, whereas *AtHMA5–AtHMA8* and *OsHMA4–OsHMA9* comprised the Cu/Ag subgroup (Williams and Mills, 2005). *PtHMA1* and *PtHMA4* belonged to the Zn/Co/Cd/Pb subgroup, whereas the rest of the genes were classified into the Cu/Ag subgroup (Figure 2). The number of genes in the *Populus* (10 genes) Cu/Ag subgroup was significantly higher than that in rice (6 genes) and *Arabidopsis* (4 genes).

### Chromosomal location and gene duplication of *Populus* HMAs

**Figure 3:**
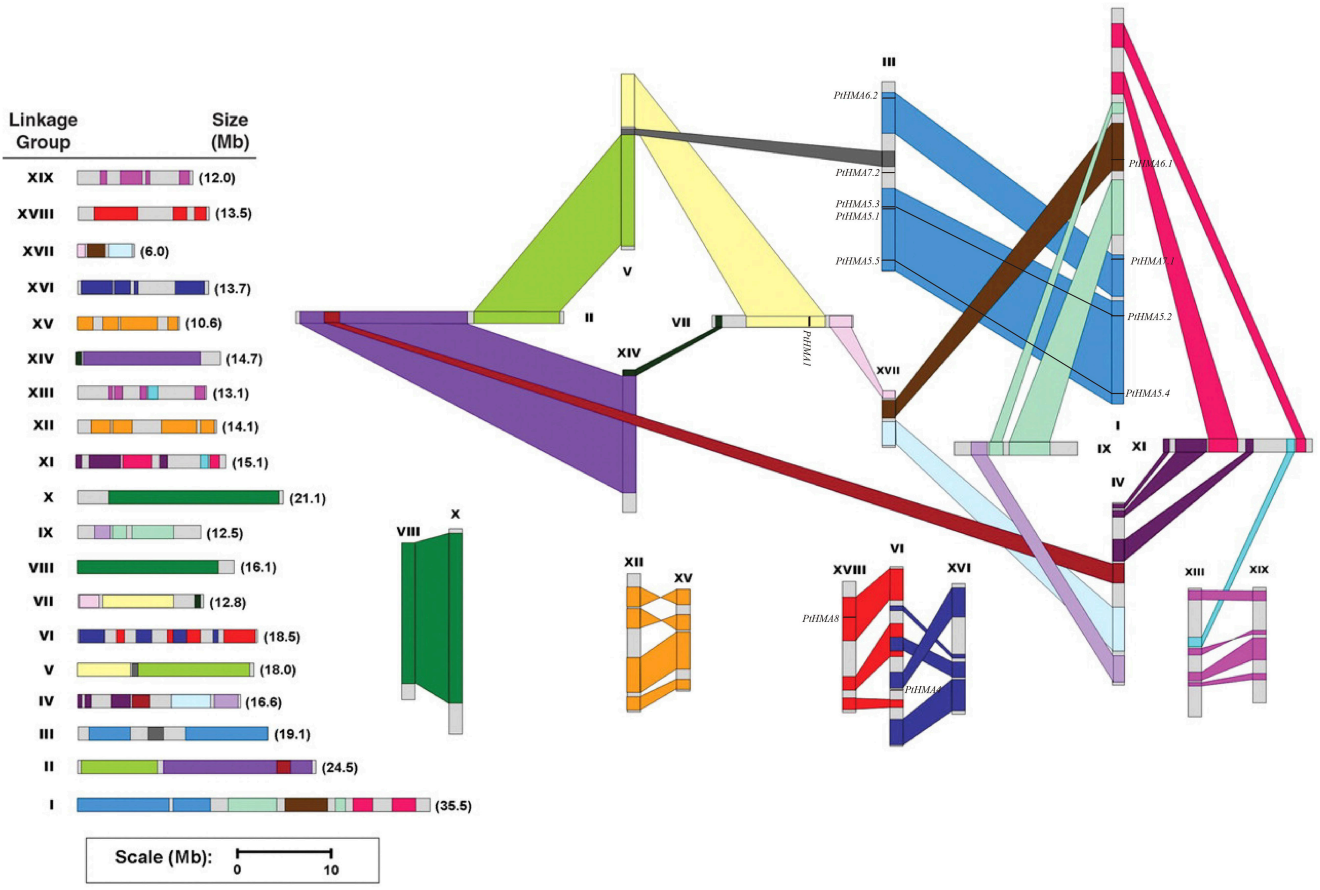
**Chromosomal location of ***Populus*** HMA genes**. A total of 12 HMA genes are mapped to the 19 linkage groups (LG). A schematic view of chromosome reorganization by recent whole-genome duplication in *Populus* is shown. Segmentally duplicated homologous blocks are indicated with the same color. The *scale* represents megabases (Mb). The LG numbers are indicated at the top of each bar.

**Figure 4:**
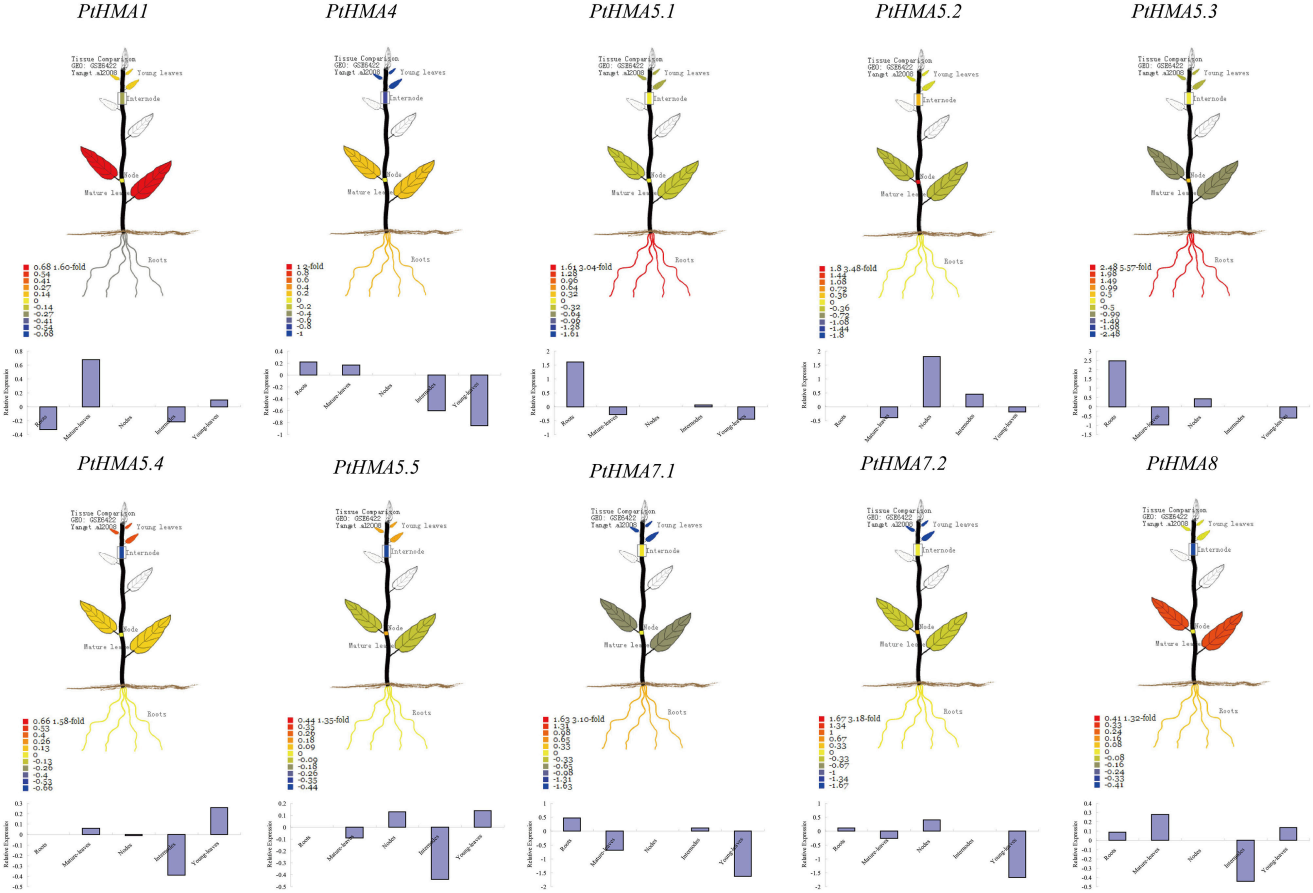
**Organ-specific expressions of ***PtHMA*** genes in mature-leaves, young-leaves, roots, nodes, and internodes**. The visual images were searched from PopGenIE v2.0. Each gene is indicated in every graph.

In *silico* mapping of gene loci showed that all 12 *Populus* HMA genes were physically located on 19 linkage groups (LG). A previous study revealed that the *Populus* genome has undergone genome-wide duplications, followed by multiple segmental duplications, tandem duplications, and transposition events (Hu et al., 2012). *Populus* HMA genes were differentially expressed in various tissues (**Figure 4**). The *PtHMA* genes were located on chromosomes I, III, VI, VII, and XVII. Chromosome III harbored the highest number of HMA genes (five), followed by chromosome I (four). Only one HMA gene was detected on each of chromosomes VI, VII, and XVII (Figure 3).

The segmental duplication associated with the salicoid duplication event that occurred 65 million years ago largely contributed to the expansion of several multi-gene families (Kalluri et al., 2007; Barakat et al., 2009, 2011; Wilkins et al., 2009; Hu et al., 2010). We used the duplicated blocks established in a previous study (Tuskan et al., 2006) to confirm the possible relationship between HMA genes and potential segmental duplications. The distribution of HMA genes relative to the corresponding duplicate blocks is illustrated in Figure 3. In this study, of the 12 *Populus* HMA genes, 10 were located in duplicated regions and two were located outside duplicated blocks. Among these 10 genes, four were preferentially retained duplicates that were located in both duplicated regions, whereas the others were only in one of the blocks and lacked duplicates in the corresponding block. These results indicated that duplication events and tandem repeats contributed to the expansion of the HMA gene family in the *Populus* genome.

### Promoter *cis*-elements analysis

Phytohormones such as salicylic acid (SA), jasmonic acid (JA), ethylene (ET), and abscisic acid (ABA) enable plants to adapt to abiotic stresses (Fujita et al., 2006; Santner and Estelle, 2009). We identified putative *cis*-acting regulatory DNA elements (Table 2) on the basis of the obtained promoter sequences (2000 bp). The *PtHMA* gene family promoter sequences showed several *cis*-elements that were related to phytohormones and environmental stress signal responsiveness. Most of the 12 HMA genes contained *cis*-elements such as ABA responsive (ABREs and AREs), heat stress responsive (HSEs), MYB binding site involved in drought-inducibility (MBS), and defense and stress responsive TC-rich repeat elements. *PtHMA1, PtHMA4, PtHMA5.1, PtHMA5.2, PtHMA6.2, PtHMA7.1*, and *PtHMA8* contained the *cis*-element TCA-element, which is involved in salicylic acid responsiveness. SA appears to be an effective therapeutic agent in plant responses to biotic and abiotic stresses (Rivas-San Vicente and Plasencia, 2011). It was also reported that SA induced adaptability to Cd, Hg, Ni, Pb, and Mn toxicity (Mostofa and Fujita, 2013). The CGTCA-motif or TGACG-motif involved in MeJA-responsiveness was identified in seven genes (Table 2). Moreover, *PtHMA1* and *5.4* contained the *cis*-acting regulatory element, as-2-box, and as1, which are involved in shoot-specific and root-specific expression, respectively. *PtHMA5.5* contained a CAT-box that is related to meristem expression.

**Table 2:** **The ***cis***-elements of HMA gene promoter in ***P. trichocarpa*** that are related to stress**.

**Gene name**	**cis-elements related to abiotic stress**
*PtHMA1*	ABRE ARE HSE MBS CGTCA-moti TGACG-motif TCA-element as1 as-2-box
*PtHMA4*	ABRE ARE HSE TCA-element TC-rich repeats WUN-motif EIRE
*PtHMA5.1*	ABRE ARE HSE MBS TGACG-motif TCA-element TC-rich repeats
*PtHMA5.2*	ABRE ARE HSE MBS ERE TCA-element CCAAT-box
*PtHMA5.3*	ABRE ARE HSE MBS ERE CGTCA-motif TGACG-motif TC-rich repeats
*PtHMA5.4*	ARE HSE MBS ERE CGTCA-motif TGACG-motif TC-rich repeats WUN-motif as1 as-2-box
*PtHMA5.5*	ABRE HSE MBS ERE CAT-box
*PtHMA6.1*	ABRE ARE HSE MBS CGTCA-motif TGACG-motif TC-rich repeats
*PtHMA6.2*	ABRE ARE HSE MBS CGTCA-motif TGACG-motif TCA-element TC-rich repeats
*PtHMA7.1*	ABRE ARE HSE TCA-element TC-rich repeats
*PtHMA7.2*	ABRE HSE MBS ERE CGTCA-motif TGACG-motif TC-rich repeats
*PtHMA8*	ARE HSE MBS ERE TCA-element TC-rich repeats

### Tissue-specific expression profile in PopGenIE v2.0

The expression profiles of 10 HMA genes from the Affymetrix (GSE6422) microarray data showed specific expression patterns in different tissues (Figure 4). Two HMA genes (*PtHMA6.1* and *PtHMA6.2*) did not have corresponding microarray sequences in the database, which suggested that the two genes were pseudogenes. Alternatively, the two genes showed a low level of transcript abundance or had special temporal and spatial expression patterns that were not examined in the libraries. The microarray data showed high expression levels of *PtHMA1* and *PtHMA4* in leaves. We also observed high expression levels of *PtHMA4* in the roots. Cu/Ag subgroup members (*PtHMA5.1–PtHMA8*) also showed higher expression levels in the roots than in other tissues. *PtHMA5.4* and *PtHMA5.5* had higher expression levels in young leaves than in other tissues. In addition, in promoters of *PtHMA5.4* and *PtHMA5.5*, the genes were related to meristem expression.

### Expression of HMA genes under different heavy metal stresses

**Figure 5:**
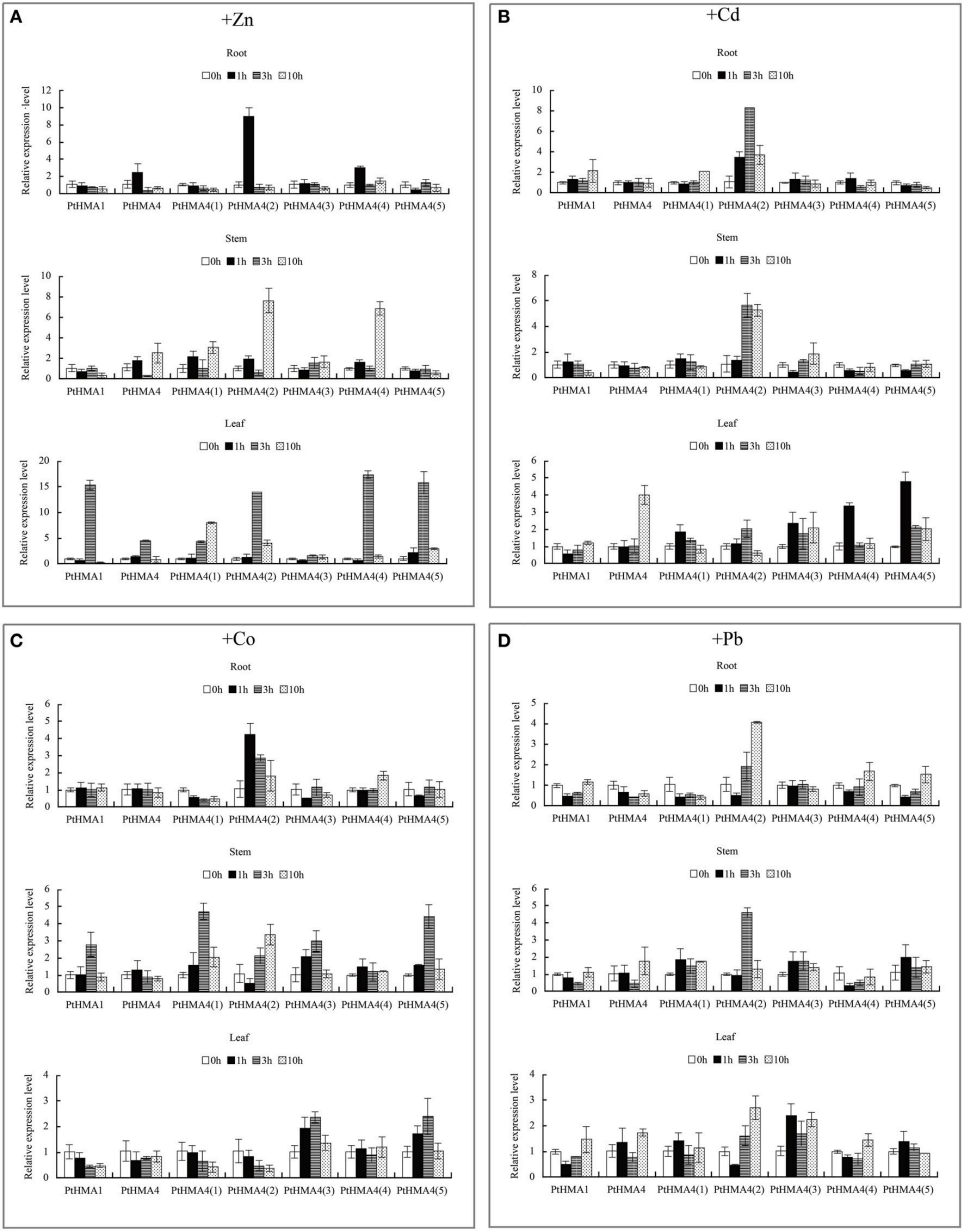
**Expression profiles of the ***PtHMA*** genes under heavy metal conditions**. QRT-PCR was used to analyze the expression patterns of 7 *PtHMA* genes in the roots, stems, and leaves under excessive amounts of Zn **(A)**, Cd **(B)**, Co **(C)**, and Pb **(D)**. *X-axis* represents different genes. The *y-axis* represents relative gene expression levels normalized to the *Populus* reference gene, actin. Standard deviations were derived from three replicates of each experiment.

We studied the expression profiles of *PtHMA* genes in *P. trichocarpa* under heavy metal stress (Figure 5). The genes *PtHMA1* and *PtHMA4* and its five alternatively spliced genes (Zn/Co/Cd/Pb subgroup) were exposed to Zn, Cd, Co, and Pb stresses. Under Cd stress, the expression of *PtHMA*1 gradually increased in the roots compared to the non-treated samples, the expression level increased in the stem after 1 h of treatment, and the expression level of *PtHMA1* in the leaves gradually increased with treatment time (Figure 5B). Therefore, *Populus HMA1* might not only be involved in Zn transport, but also in Cd transport from the roots to the leaves. The expression level of the *PtHMA4(2)* gene was higher compared to that of the other alternatively spliced genes [*PtHMA4(1), PtHMA4(3), PtHMA4(4)*, and *PtHMA4(5)*] under excess Zn, Cd, Co, and Pb stresses. In the 200 μM ZnSO_4_ treatment, *PtHMA4(2)* was upregulated at 1 h in the roots and 10 h in the stems compared to that observed in the roots and leaves, and the expression in the roots peaked at 3 and 10 h of Zn treatment (Figure 5A). The transcription levels of *PtHMA4(2)* were higher in the roots and stems than that in the leaves with Cd treatment at all-time points. Moreover, *PtHMA4(2)* was upregulated in the roots, stems, and leaves after exposure to excessive Pb (Figure 5D), and in the roots and leaves with exposure to high amounts of Co (Figure 5C).

**Figure 6:**
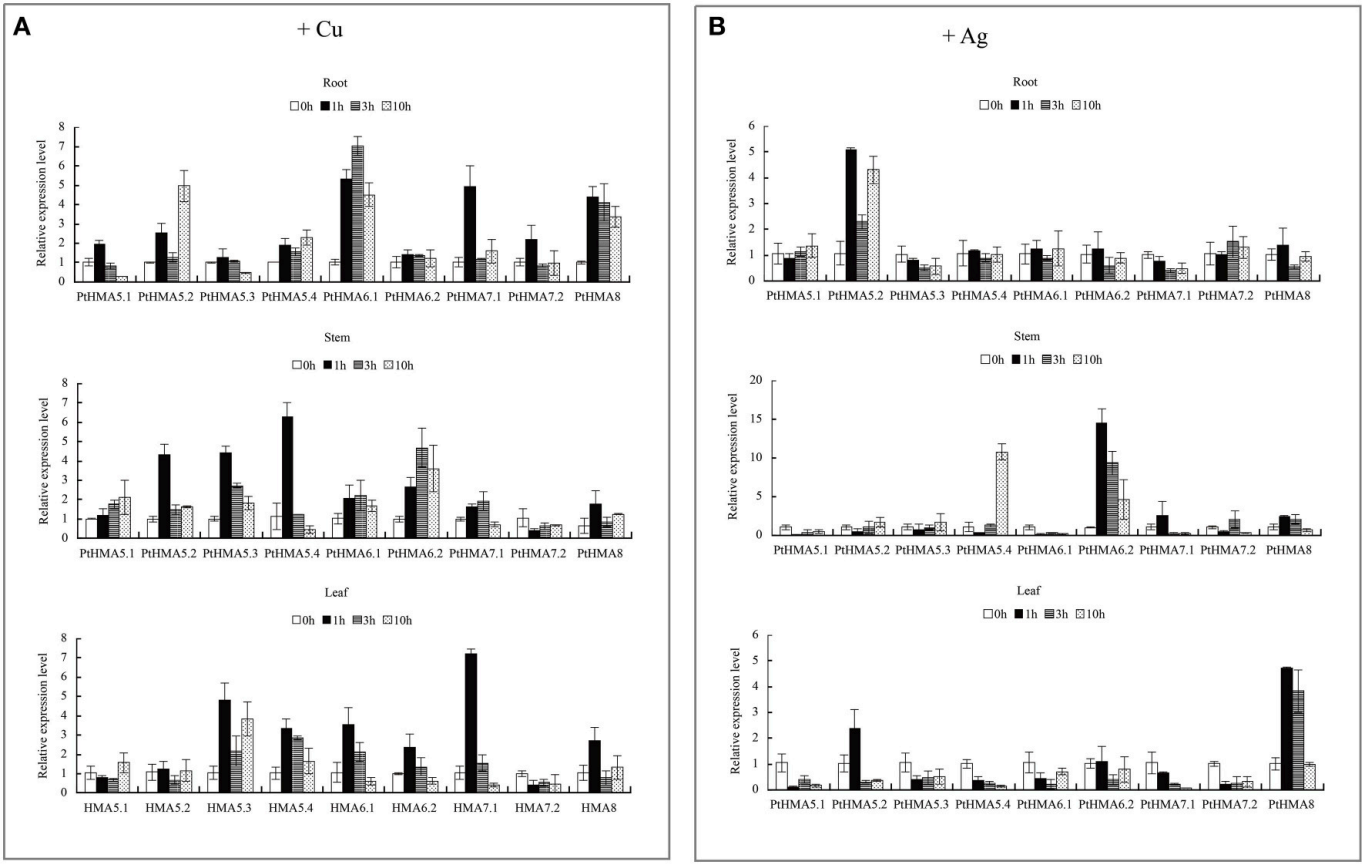
**qRT-PCR was performed to analyze the expression patterns of nine ***PtHMA*** genes in the roots, stems, and leaves under excessive amounts of Cu (A) and Ag (B)**. *X-axis* represents different genes. The *y-axis* represents relative gene expression levels normalized to the *Populus* reference gene, actin. Standard deviations were derived from three replicates of each experiment.

We further evaluated the expression patterns of *PtHMA5–PtHMA8* of the Cu/Ag subfamily. The number of genes for *PtHMA5* (5 genes), *PtHMA6* (2 genes), *PtHMA7* (2 genes) was higher than those of its alternatively spliced *Arabidopsis* counterparts. We also examined the expression of nine candidate genes subjected to excessive Cu and Ag treatments (Figure 6). The genes *PtHMA5.2, 5.4, 6.1, 7.1*, and *8* were unregulated in response to Cu treatment, whereas *PtHMA5.1* and *5.3* were downregulated in the roots (Figure 6A). However, the expression of five genes (i.e., *PtHMA5.2, 5.4, 6.1, 7.1*, and *8*) in the roots remained unaffected with Cu treatment (Figure 6A). On the other hand, *PtHMA5.2, 5.3*, and *5.4* were considerably upregulated in poplar stems after exposure to Cu for 1 h, whereas *PtHMA 6.2* was upregulated at all time points, with the highest expression detected after 3 h. In leaves, *PtHMA5.3, 5.4, 6.1, 6.2*, and *7.1* showed considerably high expression levels after 1 h of exposure to excessive levels of Cu (Figure 6A). The gene expression patterns of the four *Populus PtHMA5* genes were divided into two groups based on the time point when the highest transcript levels were observed in different tissues and organs (Figure 6A). One group (*PtHMA5.1* and *PtHMA5.3*) accumulated the highest transcripts in the roots at 1 h after Cu treatment, whereas that of the other groups, *PtHMA5.2* and *PtHMA5.4*, exhibited two peaks, namely, at 1 and 10 h, of Cu treatment. The expression level of *PtHMA5.1* in the stems and leaves gradually increased with treatment time. The highest transcription levels for *PtHMA5.2, 5.3*, and *5.4* were observed in the roots at 3 and 10 h of excessive Cu treatment. *PtHMA6.1* and *PtHMA6.2* were highly expressed in the roots but were also expressed in stems and leaves. These results suggest that *PtHMA6.1* and *PtHMA6.2* play a role in Cu transport during plant growth and development. The expression of *PtHMA7.1, PtHMA7.2*, and *PtHMA8* was upregulated in the roots, stems, and leaves, and *PtHMA7.1* was considerably upregulated in the leaves (Figure 6A). The expression level of *PtHMA8* in the roots was higher than that in the stems and leaves. In the roots, *PtHMA5.1, 5.2*, and *7.2* were upregulated in response to Ag treatment, whereas the other genes remained unaffected at all-time points. In the stems, *PtHMA5.4, 6.2*, and *8* showed a significant increase in transcript levels at 10 h, and *6.2* presented a higher expression level at all-time points compared to that of the control (Figure 6B). Ag treatment increased the expression level of the *PtHMA5.2, PtHMA5.4, PtHMA6.2*, and *PtHMA8* transcripts compared to the five other HMA genes (*PtHMA5.1, PtHMA5.3, PtHMA6.1, PtHMA7.1*, and *PtHMA7.2)*. The expression of *PtHMA5.4* and *PtHMA6.2* in the stems peaked at 10 and 1 h after excess Ag treatment, respectively. *PtHMA8* was expressed in the entire plantlet, whereas its expression level was higher in the leaves than in the stems and roots under excess Ag stress. In contrast, *PtHMA6.2* and *PtHMA8* had the highest levels of transcript abundance in the stems and leaves relative to those of the other HMA genes with Ag treatment.

**Figure 7:**
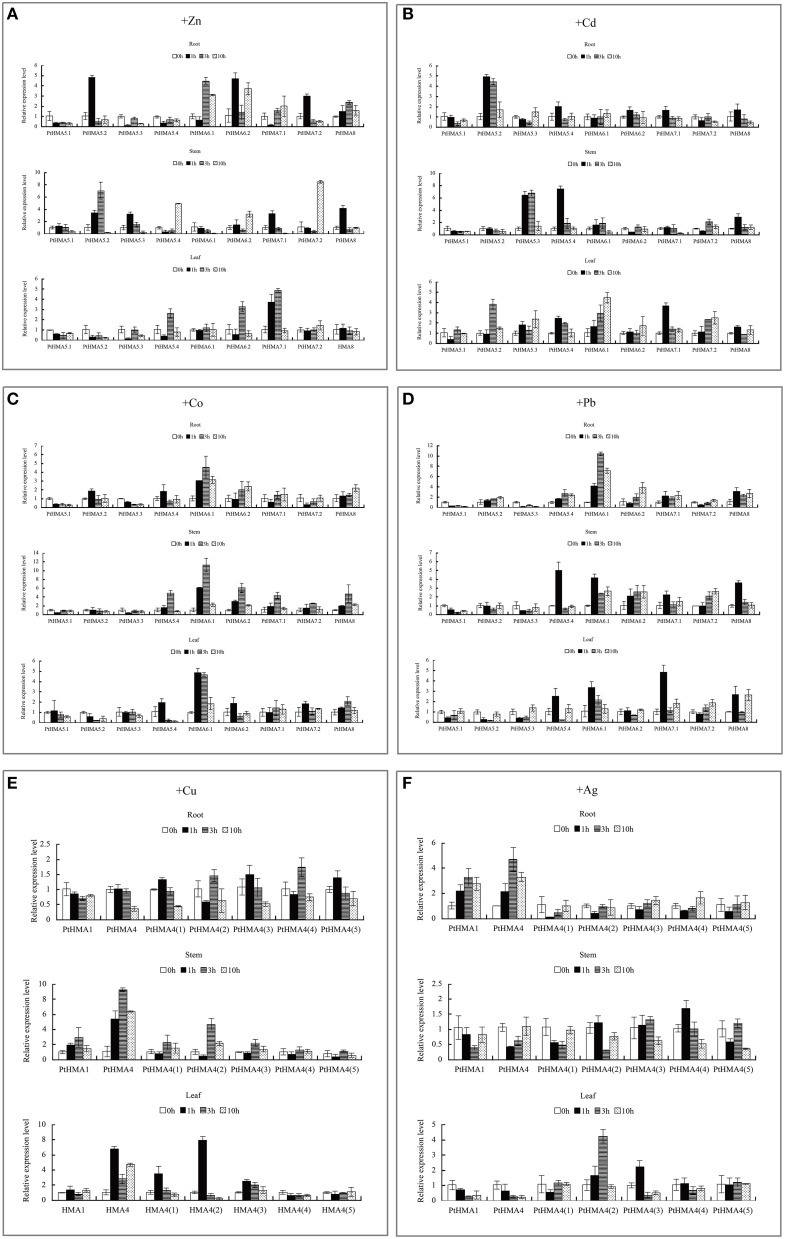
**qRT-PCR was used to analyze the expression patterns of nine ***PtHMA*** genes in the roots, stems, and leaves under excessive amounts of Zn (A), Cd (B), Co (C), and Pb (D), and seven ***PtHMA*** genes in the roots, stems, and leaves under excessive amounts of Cu (E) and Ag (F)**. *X-axis* represents different genes. The *y-axis* represents relative gene expression levels normalized to the *Populus* reference gene, actin. Standard deviations were derived from three replicates of each experiment.

**Figure 8:**
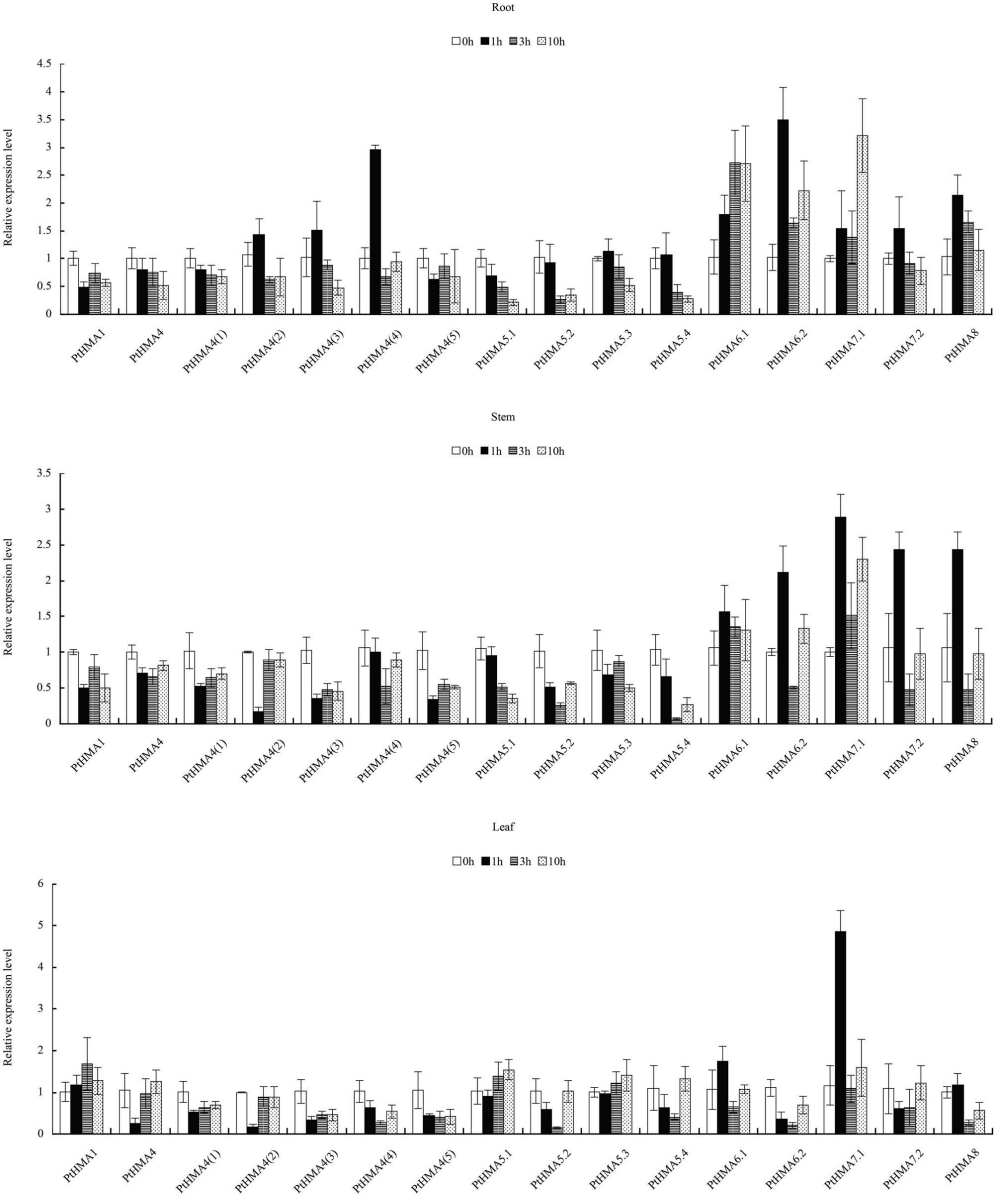
**qRT-PCR was performed to analyze the expression patterns of 16 ***PtHMA*** genes in the roots, stems, and leaves under excessive Mn stress**. *X-axis* represents different genes. The *y-axis* represents relative gene expression levels normalized to the *Populus* reference gene, actin. Standard deviations were derived from three replicates of each experiment.

Our qRT-PCR data further showed that *PtHMA5–PtHMA8* might have also been induced by Zn, Cd, Co, or Pb. Of these genes, two homologous genes (*PtHMA6.1* and *PtHMA6.2*) exhibited particularly high transcript accumulations in the roots, stems, and leaves after exposure to excessive amounts of Zn, Co and Pb, especially the roots (Figure 7). The expression level of *PtHMA5.2, PtHMA5.3, PtHMA5.4, PtHMA6.1, PtHMA7.1*, and *PtHMA7.2* increased in different tissues under excessive Cd stress. The expression of *PtHMA6.1* was higher in the leaves than in the roots and stems (Figure 7B). The *PtHMA1–PtHMA4* phylogenetic cluster with the Zn/Cd/Co/Pb subclass of HMAs (Figure 2) was possibly induced by Cu or Ag. For example, *PtHMA4* and *PtHMA4(2)* were mainly expressed in the stems under excessive Cu stress (Figure 7E). Also, *PtHMA1* and *PtHMA4* were upregulated in the roots compared to that observed in the stems and leaves after excessive Ag stress (Figure 7F). Mn is an important micronutrient that is required in various stages of plant growth and development. Five genes (*PtHMA6.1, PtHMA6.2, PtHMA7.1, PtHMA7.2*, and *PtHMA8*) showed higher transcript levels in the roots, stems, and leaves under Mn stress, with peak expression at 1 and 10 h of excess Mn treatment (Figure 8).

## Discussion

### The HMA gene family in *Populus*

In the present study, we identified 12 full-length HMA genes and five alternatively spliced genes of *PtHMA4* in the *P. trichocarpa* genome, each of which contains at least one HMA motif. The lengths of these sequences significantly varied, implying a high degree of complexity among the HMA genes. Furthermore, WoLF PSORT analyses allowed us to putatively localize the genes. However, experimental validation may be required for a more accurate localization. Preliminary analysis of the HMA gene family was performed in the plant models, *Arabidopsis* and rice (Williams and Mills, 2005; Takahashi et al., 2012). These studies revealed that *AtHMA1, AtHMA6*, and *AtHMA8* were located in the chloroplast; *AtHMA2, AtHMA4*, and *AtHMA5* were located in plasma membrane; and *AtHMA3* and *AtHMA7* were in the vacuole and Golgi, respectively (Williams and Mills, 2005). The present study showed that most of the HMA genes were predicted as plasma membrane proteins, except *PtHMA1* and *PtHMA5.1*. *PtHMA1* was located in the cytoplasm, though it was placed in the same group as *PtHMA4* (Zn/Cd/Co/Pb). *PtHMA5.1* was also located in the cytoplasm, which differed from the other members of *PtHMA5* (i.e., *PtHMA5.2–PtHMA5.5*). These results indicated that the same phylogenetic grouping based on sequence similarity did not necessarily correspond to the same subcellular localization. Therefore, homologous genes may show differences in gene function and signal transduction. Although our preliminary prediction showed that *PtHMA1* and *PtHMA5.1* were located in the cytoplasm, we could not determine the specific organelle which the genes were associated with. Further studies may help to determine the exact location of these genes. However, subcellular localization of these genes in *Populus* might change with varying metal supply, as seen in the mammalian Wilson's disease protein, ATP7B, which moves from the Golgi to the plasma membrane at higher metal concentrations (Petris et al., 1996). We speculate that the localization of HMA family proteins changes with differences in metal supply and utilize specific mechanisms with various metal stresses.

It has been reported that exon-intron increase or decrease can be caused by the integration and realignment of gene fragments (Xu et al., 2012). Therefore, structural gene variation plays a major role in the evolution of gene families (Xu et al., 2012). In the evolutionary history of *Populus*, members of the *HMA* gene family underwent rigorous selection. The structure of *Populus HMA* genes is well conserved, and these genes have different numbers of exons. Although *PtHMA5–8* belonged to the same phylogenetic group (group Zn/Cd/Co/Pb), their number of exons varied. *PtHMA6.1* and *PtHMA6.2* had more exons than the others, which in turn may be expressed in specific tissues.

### Comparative analysis of the HMA genes in *Populus, Arabidopsis*, and rice

In *Arabidopsis*, the Cu subgroup has three domains, E1-E2 ATPase domains (PF00122), a heavy metal-associated domain (PF00403), and a haloacid dehalogenase-like hydrolase (PF00702). The Zn subgroup has two domains, namely, the E1-E2 ATPase and haloacid dehalogenase-like hydrolase (Table S3). Unlike *Arabidopsis*, the HMAs of *P. trichocarpa* has similar domains. However, phylogenetic analysis showed that *PtHMA5.5* had only an E1-E2 ATPase and a haloacid dehalogenase-like hydrolase domain compared to the other members of its subgroup. Our analysis also revealed that the number of *Populus, Arabidopsis*, and rice HMA genes varied in most subfamilies. For example, *Arabidopsis* and rice just have one of each of the *HMA5, HMA6, HMA7* genes. However, *Populus* has five members of *HMA5* and two members of *HMA6* and *HMA7*. *Populus* lacks *HMA2* and *HMA3*, but alternative splicing of *PtHMA4* may occur and compensate for the two missing genes. Therefore, the present study provides a platform for future studies on the role of HMA genes in *Populus*.

### Chromosomal location and gene duplication

Tuskan et al. (2006) suggested that the *Populus* genome underwent at least three rounds of genome-wide duplication, followed by multiple segmental duplication, tandem duplication, and transposition events such as retroposition and replicative transposition. Gene duplication events, including tandem and segmental duplication, play an important role in genomic expansions and realignments (Vision et al., 2000; Kumar et al., 2011). In the present study, 5/6 of the *Populus* HMA genes were located in duplicated regions, whereas only two genes were located outside the duplicated blocks. These results suggest that dynamic rearrangements might have occurred following the segmental duplication, which led to the loss of some genes. These findings also showed that *PtHMA5.2* and *PtHMA5.3*, and *PtHMA5.4* and *PtHMA5.5* belonged to the same branch of the phylogenetic tree and were present in homologous regions of chromosomes I and III, respectively (Figure 3). This finding might explain the homology of the two genes. The tandem duplications might have impacted the expansion of the HMA gene family. Our results also showed that only one pair (*PtHMA5.1/5.3*) of the HMA genes underwent tandem duplication, as indicated by the >90% similarity in amino acid sequence. In comparison, two segmental duplications (*PtHMA5.2*/*5.3* and *PtHMA5.4*/*5.5*) were identified. These results corroborate the findings of previous studies involving other gene families (Kalluri et al., 2007).

### Transcript profiles of HMA genes in *Populus*

Plant metal homeostasis must be tightly regulated to ensure sufficient micronutrient (e.g., Zn, Cu, and Fe) supply to different organs, and to prevent non-essential metals (e.g., Cd and Pb) to reach toxic levels that may result in deleterious effects (Hall, 2002). Metal transporters play important roles in various aspects of essential and toxic metal distribution in plants. All members of HMAs in *A. thaliana* are functionally characterized (Deng et al., 2013). *AtHMA1* and *OsHMA1* are involved in Zn transport (Kim et al., 2009). *AtHMA1* transports Zn, Cu, and Ca (Williams and Mills, 2005; Seigneurin-Berny et al., 2006; Kim et al., 2009). However, the present study did not indicate transcript accumulation of *PtHMA1* under excessive Cu stress. Our data showed that the expression patterns of *PtHMA1* and *AtHMA1* were not similar. The *HMA1* in *Populus* not only transported Zn and Cu but also Cd from the roots to the leaves. *AtHMA4* is induced by Cd and Zn and is expressed in tissues surrounding the vascular vessels of the root vascular (Mills et al., 2003). In this study, in addition to Cd and Zn, *PtHMA4(2)* was also induced by Pb and Co. The transcription levels of *PtHMA4(2)* were almost unregulated in the roots, stems, and leaves (Figure 5). The above results indicated differential expression patterns for various *PtHMAs* in the roots, stems, and leaves, thus confirming tissue-specific expression. These findings may have significant implications in *Populus. AtHMA5* is involved in the Cu translocation from the roots to the shoots or Cu detoxification of roots (Kobayashi et al., 2008). *AtHMA6, AtHMA7*, and *AtHMA8* also play an important role in transport Cu (Woeste and Kieber, 2000; Shikanai et al., 2003; Abdel-Ghany et al., 2005; Catty et al., 2011). *OsHMA5* is involved in loading Cu to the xylem of the roots and other organs (Deng et al., 2013). The results of the present study suggested that the role of *PtHMA5* may differ from that of *AtHMA5* and *OsHMA5. AtHMA5* is involved in Cu detoxification, whereas *OsHMA5* is responsible for xylem loading of Cu. However, *PtHMA5* plays an important role in Ag detoxification in addition to Cu detoxification. *PtHMA6.1, PtHMA6.2, PtHMA7.1, PtHMA7.2*, and *PtHMA8* are similar to their homologs in *Arabidopsis* and function in transporting or detoxifying Cu. *PtHMA6.2* and *PtHMA8* had higher transcription levels under Ag stress. *PtHMA6.1, PtHMA6.2, PtHMA7.1, PtHMA7.2*, and *PtHMA8* were induced by Mn in the roots, stems, and leaves, suggesting that *PtHMA* genes help in scavenging excess Ag and Mn. The considerable differences in expression among *PtHMA* genes suggest that these genes perform varying physiological and biochemical functions to adapt to complicated challenges.

Metal transporters play important roles in various aspects of essential and toxic metal distribution in plants. Moreover, under different metal stress, the expression of *PtHMAs* varied in the roots, stems, and leaves, thereby confirming tissue-specific expression. In summary, the present study provides a theoretical basis to select metal-specific genes for related functional validation. Expression studies of *PtHMA* genes suggest their special roles during the plant growth and developmental stages. Their functions are similar to that in rice or *Arabidopsis*, although more complicated. The results of the present study led to a better understanding of the *Populus* phenotype and help to lay out the first step toward engineering a hyper accumulating *Populus* phenotype that may be utilized in phytoremediation. However, the specific functions of the most *PtHMA* genes remain unknown. Further experiments, such as Western blotting should be carried out to demonstrate that those HMA genes accounting for the responses to metal stress are real. Also, ectopic expression of *PtHMA* genes in *Arabidopsis* or other plant species is necessary to investigate heavy metal accumulation or tolerance phenotypes of the transgenic plants in response to heavy metal treatments. It would improve on the results of this study.

## Author contributions

Conceived and designed the experiments: DL, CL. Performed the experiments: DL, HZ. Analyzed the data: DL, XH, XX, and CL. Contributed reagents/materials/analysis tools: DL, QL, ZW, HW, HL, MW, ZW, and CL. Wrote the paper: DL, CL. All authors approved the final manuscript.

### Conflict of interest statement

The authors declare that the research was conducted in the absence of any commercial or financial relationships that could be construed as a potential conflict of interest.

## Supplementary material

Figure S1 **Phylogenetic tree of the HMA protein families in ***P. trichocarpa*** was constructed based on the alignments of the full-length HMA protein sequences**. The tree was generated using the MEGA5 program with the neighbor-joining method. For statistical reliability, bootstrap analysis was conducted with 1000 replicates.

Table S1 **The primers of ***PtHMA*** genes employed in qRT-PCR analysis**. F represents the forward primer, whereas R represents the reverse primer.

Table S2 **Motif sequences of the HMA genes identified in ***Populus trichocarpa*****.

Table S3 **Domains of the HMA genes in ***Arabidopsis thaliana*** and ***P. trichocarpa*****.
