# Supplementary figures and images for: Genome-Wide Analysis and Heavy Metal-Induced Expression Profiling of the HMA Gene Family in Populus trichocarpa
Source: Front Plant Sci. 2015 Dec 23;6:1149. doi: 10.3389/fpls.2015.01149 (PMC4688379; doi:10.3389/fpls.2015.01149)

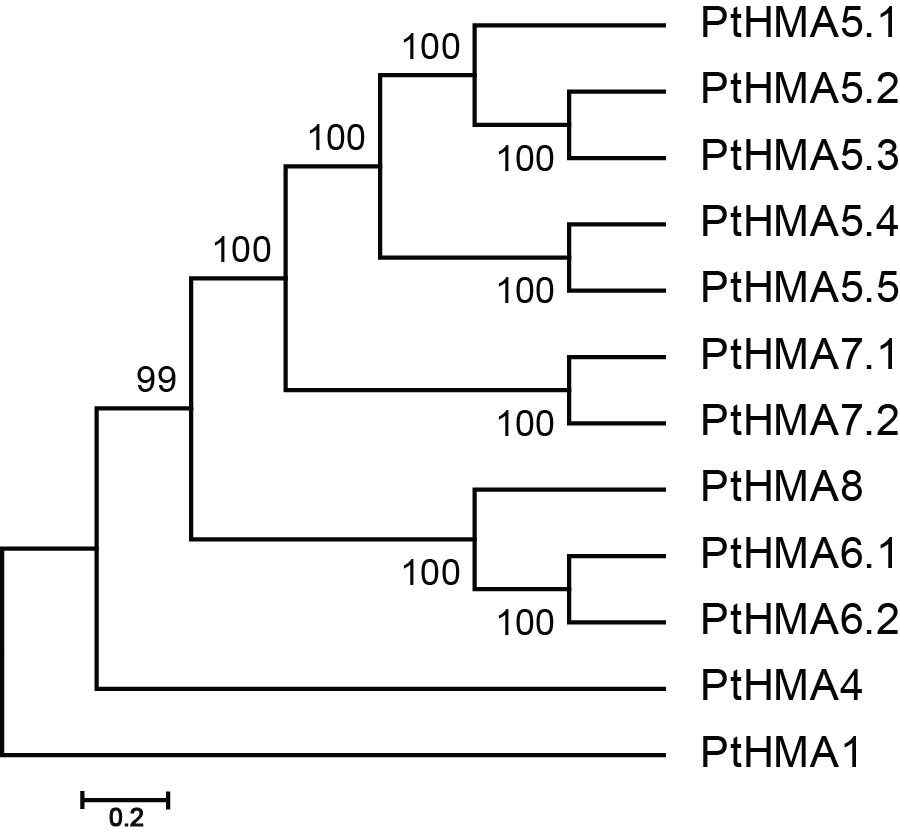

Supplement: Figure S1 — Phylogenetic tree of the HMA protein families in P. trichocarpa was constructed based on the alignments of the full-length HMA protein sequences. The tree was generated using the MEGA5 program with the neighbor-joining method. For statistical reliability, bootstrap analysis was conducted with 1000 replicates. [file Image1.JPEG]
