# Supplementary material for: Genome-Wide Analysis and Heavy Metal-Induced Expression Profiling of the HMA Gene Family in Populus trichocarpa
Source: Front Plant Sci. 2015 Dec 23;6:1149. doi: 10.3389/fpls.2015.01149 (PMC4688379; doi:10.3389/fpls.2015.01149)
Supplement: Table S1 — The primers of PtHMA genes employed in qRT-PCR analysis. F represents the forward primer, whereas R represents the reverse primer. [file Table1.DOC]

**Table S1** The primers of PtHMA genes for RT-qPCR. F represents forward primer while R represents reverse primer.

| Gene name | Primer (5'-3') |
| --- | --- |
| *PtHMA1* | F CTCACTCTCTGCTGCTGCTAAT |
|  | R GGGAGGATGAGAGAGGGAATTG |
| *PtHMA4* | F CTGCTTTTACGAGGCAGCAAATC |
|  | R CCCACATTCCACCTTCTTGACAT |
| *PtHMA4(1)* | F ATTGAGAAGGTTCAGAGCG |
|  | R TCACAACTTTCTGGCTAGAG |
| *PtHMA4(2)* | F AAGCTGAGAAGGTTCAGAGCG |
|  | R GTTCAAGCCTTGCCTCAGGATG |
| *PtHMA4(3)* | F CTCAGGGTGTTGCTCTAGACC |
|  | R GATTGGCATCCTGAGGCAAGGC |
| *PtHMA4(4)* | F CGGGATGGTGTTGCTCTAGAC |
|  | R GGTCTAGAGCAACACCCTGAG |
| *PtHMA4(5)* | F GCTGAGGTGAAGCTTGTGGCCC |
|  | R GGTCTAGAGCAACACCCTGAGTC |
| *PtHMA5.1* | F GCATCTCAAGGTGTTCTAATCAAAG |
|  | R AAGGGTGCTCACTATTCATCTCA |
| *PtHMA5.2* | F AGAAAGTTATGGGGACTTATCGCC |
|  | R GTATTCCAGGAAGCCTTTTGACAG |
| *PtHMA5.3* | F ACGATACAGGATTTGAAGCCATAC |
|  | R TCTACACTTTGAACACCAGGGAG |
| *PtHMA5.4* | F GAGAAGCTGGTCTTTACCCAAAAC |
|  | R CTTTGATGAGCACACCTTGAGAAG |
| *PtHMA6.1* | F TGTACCCAACTGGCAGAAAGAG |
|  | R ACATGACCAAGGAGGCATACAG |
| *PtHMA6.2* | F CCATTTCAAGAAGTGGAGG |
|  | R CTGTCACCAGACAACATGTAC |
| *PtHMA7.1* | F TCCAAAGATGTTGGAGAGACCT |
|  | R CACCCATTAAGAAGGGTCCACA |
| *PtHMA7.2* | F CTCTCAAGAAAAACCTTCTCTCGC |
|  | R CTAGGGAAGGAAAGAACATCCCAG |
| *PtHMA8* | F TGTATCTGCAGCAACTTTTGCC |
|  | R GCGATGTTCCAACTAGGATTGC |
| *Actin* | F GAAGTCCTCTTCCAGCCTTCTC |
|  | R CTTGATCTTCATGCTGCTTGGG |
