# Supplementary material for: Genome-Wide Analysis and Heavy Metal-Induced Expression Profiling of the HMA Gene Family in Populus trichocarpa
Source: Front Plant Sci. 2015 Dec 23;6:1149. doi: 10.3389/fpls.2015.01149 (PMC4688379; doi:10.3389/fpls.2015.01149)
Supplement: Table S2 — Motif sequences of the HMA genes identified in Populus trichocarpa. [file Table2.DOC]

***Table S2*** *Motif sequences of HMA genes identified in P.* trichocarpa

| Motif | Width(aa) | Best possible match |
| --- | --- | --- |
| 1 | 50 | IDLSRKTMSRIRQNYWWAMGYNIIGIPIAAGMLFPFTGFRLPPWIAGACM |
| 2 | 41 | ISVMVIACPCALGLATPTAVMVGTGVGASQGVLIKGGNALE |
| 3 | 50 | GTINWNGYLHIKATRVGSESALSQIVRLVEEAQMSKAPVQKFADYISKYF |
| 4 | 41 | TPVQFIPGRRFYVGSYKALRRGSPNMDVLVALGTNAAYFYS |
| 5 | 30 | LQKDGYIVAMVGDGINDSPALAAADVGMAI |
| 6 | 50 | EEEIDSRLIQRNDYIKVFPGEKVPADGFVIWGQSHVNESMITGEARPVAK |
| 7 | 45 | FFETSSMLISFILLGKYLECLAKGKTSDAMAKLMNLAPTTARLVT |
| 8 | 29 | RAHKVNYVVFDKTGTLTIGKPVVTDVRPF |
| 9 | 29 | LKSMGIRSIMVTGDNWRTAWYIAKEVGIE |
| 10 | 50 | QVCRIRVNGMTCTSCSWTVEQALQAIHGVQRAQVALATHEAEVHYDPNLL |
| 11 | 29 | EFYEWVAAAEANSEHPLAKAIVEYAKHFR |
| 12 | 41 | PLVIILSFSTWFAWFIAGIFHGYPEHWIPKNMDYFQFALQF |
| 13 | 33 | FEYHPGHGVKCTVRNKEVYVGNRKWMQEHNICI |
| 14 | 21 | GTDIAIEAADIVLMRNNLEDV |
| 15 | 41 | ENFIAEHEQMAQTGVYVAIDREVTGILYISDPLKPEAHEVI |
