# Supplementary material for: Genome-Wide Analysis and Heavy Metal-Induced Expression Profiling of the HMA Gene Family in Populus trichocarpa
Source: Front Plant Sci. 2015 Dec 23;6:1149. doi: 10.3389/fpls.2015.01149 (PMC4688379; doi:10.3389/fpls.2015.01149)
Supplement: Table S3 — Domains of the HMA genes in Arabidopsis thaliana and P. trichocarpa. [file Table3.DOC]

**Table S3** Domain of HMA genes in *Arabidopsis thaliana* and *P. trichocarpa.*

| Gene name | Domain of HMA genes |
| --- | --- |
| *AtHMA1* | E1-E2 ATPase (PF00122) haloacid dehalogenase-like hydrolase (PF00702) |
| *AtHMA2* | E1-E2 ATPase (PF00122) haloacid dehalogenase-like hydrolase (PF00702) |
| *AtHMA3* | E1-E2 ATPase (PF00122) haloacid dehalogenase-like hydrolase (PF00702) |
| *AtHMA4* | E1-E2 ATPase (PF00122) haloacid dehalogenase-like hydrolase (PF00702) |
| *AtHMA5* | E1-E2 ATPase (PF00122) haloacid dehalogenase-like hydrolase (PF00702) heavy metal-associated domain (PF00403) |
| *AtHMA6* | E1-E2 ATPase (PF00122) haloacid dehalogenase-like hydrolase (PF00702) heavy metal-associated domain (PF00403) |
| *AtHMA7* | E1-E2 ATPase (PF00122) haloacid dehalogenase-like hydrolase (PF00702) heavy metal-associated domain (PF00403) |
| *AtHMA8* | E1-E2 ATPase (PF00122) haloacid dehalogenase-like hydrolase (PF00702) heavy metal-associated domain (PF00403) |
| *PtHMA1* | E1-E2 ATPase (PF00122) haloacid dehalogenase-like hydrolase (PF00702) |
| *PtHMA4* | E1-E2 ATPase (PF00122) haloacid dehalogenase-like hydrolase (PF00702) |
| *PtHMA5.1* | E1-E2 ATPase (PF00122) haloacid dehalogenase-like hydrolase (PF00702) heavy metal-associated domain (PF00403) |
| *PtHMA5.2* | E1-E2 ATPase (PF00122) haloacid dehalogenase-like hydrolase (PF00702) heavy metal-associated domain (PF00403) |
| *PtHMA5.3* | E1-E2 ATPase (PF00122) haloacid dehalogenase-like hydrolase (PF00702) heavy metal-associated domain (PF00403) |
| *PtHMA5.4* | E1-E2 ATPase (PF00122) haloacid dehalogenase-like hydrolase (PF00702) heavy metal-associated domain (PF00403) |
| *PtHMA5.5* | E1-E2 ATPase (PF00122) haloacid dehalogenase-like hydrolase (PF00702) |
| *PtHMA6.1* | E1-E2 ATPase (PF00122) haloacid dehalogenase-like hydrolase (PF00702) heavy metal-associated domain (PF00403) |
| *PtHMA6.2* | E1-E2 ATPase (PF00122) haloacid dehalogenase-like hydrolase (PF00702) heavy metal-associated domain (PF00403) |
| *PtHMA7.1* | E1-E2 ATPase (PF00122) haloacid dehalogenase-like hydrolase (PF00702) heavy metal-associated domain (PF00403) |
| *PtHMA7.2* | E1-E2 ATPase (PF00122) haloacid dehalogenase-like hydrolase (PF00702) heavy metal-associated domain (PF00403) |
| *PtHMA8* | E1-E2 ATPase (PF00122) haloacid dehalogenase-like hydrolase (PF00702) heavy metal-associated domain (PF00403) |
